# Cortical responses to lipread and text-based audiovisual stimuli in adults with and without dyslexia

**DOI:** 10.3389/fnhum.2026.1806964

**Published:** 2026-10-23

**Authors:** Yuewei Cao, Francesco Gentile, Parisa Naseri, Linda Romanovska, Milene Bonte

**Affiliations:** 1 Maastricht Brain Imaging Center, Department of Cognitive Neuroscience, Faculty of Psychology and Neuroscience, Maastricht University, Maastricht, Netherlands; 2 Department of Psychological and Social Sciences, John Cabot University, Rome, Italy; 3 Faculty of Humanities, Education and Social Sciences, University of Luxembourg, Esch-sur-Alzette, Luxembourg

**Keywords:** audiovisual integration, developmental dyslexia, fMRI, fusiform gyrus, phonetic recalibration, superior temporal gyrus

## Abstract

Phonetic recalibration enables listeners to adjust their perception of ambiguous speech sounds based on visual contextual information. In line with a specific difficulty in forming letter-speech sound associations, adults with dyslexia have been found to show no text-based, but intact lipread-based, recalibration. The underlying neural mechanisms remain unclear however. This fMRI study examined cortical responses during text-based and lipread-based phonetic recalibration in 34 adults (18 with dyslexia, 16 typical readers). Participants were exposed to ambiguous speech sounds /a?a/ paired with disambiguating visual information (“aba”/“ada” text or lipread videos), followed by auditory-only categorization trials. Contrary to previous findings, both groups showed robust text-based and lipread-based behavioral recalibration effects, with no significant group differences between typical and dyslexic readers. Neuroimaging data did yield group differences in the underlying cortical activation. Most notably, dyslexic readers demonstrated increased bilateral fusiform activation compared to typical readers during exposure to lipread videos and ambiguous speech sounds, with left fusiform activation specifically correlating with increased phonological processing difficulties. Additionally, typical readers exhibited higher right frontal activation during text-based audiovisual stimuli. These findings suggest that while adults with dyslexia did not differ significantly from typical readers in behavioral recalibration effects, they employed different neural processing strategies. Unexpectedly, this was particularly the case for lipread audiovisual stimuli. The relatively increased fusiform and superior temporal activation to lipread audiovisual stimuli in dyslexic readers possibly relates to subtle difficulties in phonological processing of ambiguous speech signals.

## 1 Introduction

Reading is a complex, culturally acquired skill that depends on the brain's ability to integrate auditory and visual information, particularly associating speech sounds with visual symbols. This process, known as letter-speech sound integration, forms the basis of fluent reading and involves a distributed network of cortical regions, including the superior temporal gyrus (STG) and the left occipito-temporal cortex (including the fusiform gyrus) (Blau et al., 2009, 2010; Kronschnabel et al., 2014). In typical readers, these key regions enable efficient letter-sound mapping through left-lateralized pathways, essential for word decoding and reading fluency (Blau et al., 2010; Van Atteveldt et al., 2004). Neuroimaging studies further suggest that dyslexic readers struggle to engage these pathways effectively, showing decreased activation in both the STG (Blau et al., 2009, 2010; Kronschnabel et al., 2014) and left fusiform gyrus (Centanni et al., 2019; Karipidis et al., 2017; Romanovska et al., 2021) during tasks requiring letter-speech sound mappings. Letter-speech sound mappings are crucial not only for developing literacy but also for processing complex linguistic structures in adulthood (Dehaene et al., 2015; Price and Devlin, 2011). From a developmental perspective, typical readers show efficient reading that correlates with a developmental shift from dorsal pathways (e.g., inferior parietal lobe) to ventral pathways (e.g., ventral occipito-temporal cortex), which specializes in rapid, automatic word processing (Brauer et al., 2013; Romanovska and Bonte, 2021). Dyslexic readers often retain greater dorsal involvement, reflecting continued reliance on explicit decoding skills (Romanovska and Bonte, 2021).

One approach to studying short-term reading-related audiovisual plasticity is the text-based recalibration paradigm (Keetels et al., 2016; 2018; Bonte et al., 2017). In this paradigm, ambiguous speech sounds are paired with disambiguating text to examine audiovisual integration. This paradigm has two phases: audio-visual exposure blocks followed by auditory-only post-test trials. During exposure blocks, an ambiguous speech sound /a?a/ (midway between /aba/ and /ada/) is paired with text cues “aba” or “ada,” allowing the visual input to serve as an anchor and biasing participants to perceive the ambiguous sound toward the visual cue. This repeated exposure creates perceptual shifts: /a?a/ paired with “aba” text biases subsequent auditory-only perception toward /aba/, while pairing with “ada” biases perception toward /ada/ (Keetels et al., 2018, 2016). This perceptual bias, known as recalibration, thus temporarily shifts phoneme boundaries through short-term audiovisual learning (Samuel and Kraljic, 2009; Vroomen and Baart, 2011). Recalibration is adaptable across modalities and has been elicited using various visual stimuli, including lipread speech (Bertelson et al., 2003), spoken word context (Norris et al., 2003), overt speech articulation (Scott, 2016), and text (Bonte et al., 2017; Romanovska et al., 2019, 2021, 2022).

The developmental trajectory of recalibration has particular relevance for dyslexia research. A longitudinal study (Romanovska et al., 2022), examined recalibration across three annual sessions in typically reading children aged 8–11 and found age-dependent inverted-U type changes in left STG and left lateral inferior precentral gyrus activation, with an initial activity increase reaching peak activation around the second session, followed by a decline in the third session. This pattern suggests that neural activation during audiovisual processing of letters and ambiguous speech sounds is modulated by developmental factors, particularly in auditory and motor regions. Notably, the significant activation changes in the left lateral inferior precentral gyrus suggest that motor speech areas are also involved in audiovisual recalibration processes.

Children with and without dyslexia have been found to show comparable behavioral text-based recalibration effects, together with significantly reduced fMRI activation in the left fusiform gyrus during audiovisual exposure blocks in dyslexic compared to typical readers (Romanovska et al., 2021). The strength of this left fusiform gyrus activity positively correlated with children's reading and phonological skills. Additionally, increased bilateral superior temporal cortex activation was associated with less fluent letter-speech sound identification, suggesting that higher activation in these regions may reflect less automatized letter-sound associations in children with dyslexia (Romanovska et al., 2021).

While the neural basis of text-based recalibration has been studied in children with varying reading skills, research examining adults with dyslexia remains limited, and no neuroimaging study has directly compared text- vs. lipread-based recalibration in this population. Previous studies in adult typical readers have separately shown that text-based and lipread recalibration generate similar perception-related activation patterns during post-test trials in auditory areas like the left Heschl's gyrus and planum temporale (Bonte et al., 2017; Kilian-Hütten et al., 2011a,b). These studies have further shown that both text-based and lipread exposure blocks engage an overlapping network of bilateral superior temporal gyrus (STG), ventral occipito-temporal cortex (vOTC), and frontal areas, while lipread recalibration additionally engages middle temporal gyrus regions (Bonte et al., 2017; Kilian-Hütten et al., 2011a,b).

Behavioral evidence suggests modality-specific differences in adults with dyslexia: Keetels et al. (2018) found selective deficits in text-based recalibration in dyslexic readers while lipread recalibration remained intact, suggesting specific challenges with letter-speech sound processing rather than a general audiovisual integration impairment. Interestingly, however, children with dyslexia show no such differences in text-based recalibration compared to typical readers (Romanovska et al., 2019; 2021). These discrepant findings may reflect developmental differences, methodological variations, or sample-specific factors.

The present fMRI study investigates the neural mechanisms underlying text- vs. lipread-based recalibration in adults with and without developmental dyslexia. Our analysis focuses on audiovisual exposure blocks, beginning with a whole-brain examination of cortical activation, followed by targeted analysis of regions of interest (ROIs) associated with audiovisual integration and reading (based on e.g., Blau et al., 2010; Plewko et al., 2018). We assess behavioral recalibration effects during MRI scanning and conduct correlation analyses between ROI activation and participants' reading and phonological skills. Based on prior findings showing selective text-based recalibration deficits in adults with dyslexia (Keetels et al., 2018), and differential engagement of underlying brain regions in children with dyslexia (Romanovska et al., 2021), we hypothesize that dyslexic adults will show reduced text-based recalibration, together with reduced left fusiform gyrus and increased left STG activation as compared to typical readers. As for lipread recalibration, we expect comparable behavioral effects in typical and dyslexic readers (Keetels et al., 2018), an additional recruitment of middle temporal gyrus (MTG) regions as compared to text-based recalibration (Hall et al., 2005; Kilian-Hütten et al., 2011a,b), with no differential activation related to reading deficits.

## 2 Methods

### 2.1 Participants

Forty-one adult volunteers (22 typical readers, 19 with dyslexia) participated in the 3T fMRI experiment at Maastricht University. The majority of participants were university students, and all participants with dyslexia reported having received a formal diagnosis during childhood or adolescence. Data from 7 participants were excluded in total. Four participants (9.8%: three typical readers, one reader with dyslexia) were excluded due to substantial head motion during fMRI scanning. The remaining three participants (7.3%: all typical readers) were excluded due to a failure to identify an individual phoneme boundary during the pre-test, as these participants categorized all sounds without an ambiguous region, rendering the individually-tailored stimulus selection inapplicable. The final sample consisted of 34 participants including 16 typical readers (11 females; 1 left-handed; mean age: 23.38 ± 4.91 years) and 18 readers with dyslexia (12 females; 1 left-handed; mean age: 25.17 ± 6.85 years), all native Dutch speakers with no reported hearing impairments, normal or corrected-to-normal vision, and no history of diagnosed comorbid developmental or neurological disorders. All participants provided written informed consent and received compensation via bank transfer. The experiment was approved by the Ethics Committee of the Faculty of Psychology and Neuroscience, Maastricht University.

#### 2.1.1 Reading and phonological skills

All participants completed reading fluency and phonological processing tasks. Reading fluency was assessed using the Eén-Minuut-Test (EMT, Brus and Voeten, 1978), in which participants read aloud as many words as possible from a list of 116 Dutch words, arranged by increasing difficulty (based on word length, frequency, and orthographic complexity), within 60 s. Reading fluency scores were based on the number of words correctly read. Phonological processing was evaluated with the Spoonerisms test from the GL&SCHR (Depessemier and Andries, 2009), in which participants' task was to swap the initial phonemes of two words (e.g., “ruime kabine” becomes “kuime rabine”). This task included 20 items, along with a preliminary practice session of six examples. Phonological awareness was scored as accuracy-corrected reaction time (total response time divided by the number of correct responses, multiplied by 10, in seconds), with higher values indicating poorer performance.

### 2.2 Stimuli

Auditory stimuli for both text-based and lipread-based recalibration consisted of recordings of a native Dutch male speaker articulating /aba/ and /ada/ (same as Bertelson et al., 2003), with each recording lasting 650 ms. A continuum of nine tokens (BD1–BD9) from clear /aba/ to clear /ada/ was created by altering the second formant (F2) in increments of 39 Mel while keeping the duration of the stimuli constant, using PRAAT software (Boersma and Weenink, 1999; see Bertelson et al., 2003). To identify the most ambiguous /a?a/ token, participants first listened to all sounds along the continuum. The token that was identified equally often as /aba/ and /ada/ was considered the most ambiguous for that participant. This token corresponded to the participant's individual perceived phoneme boundary.

Visual stimuli for text-based recalibration involved displaying the non-words “aba” and “ada” in white, centered on a black screen in Times New Roman font for 1 second. Lipreading recalibration stimuli consisted of video recordings of lip-movements of the male speaker pronouncing “aba” or “ada”, with each video lasting 720 ms (Bertelson et al., 2003). The slight difference in duration (720 vs. 650 ms) reflects the natural offset between the acoustic and visible articulatory signal (Bertelson et al., 2003). All stimuli were presented using Presentation software (Version 18.0, Neurobehavioral Systems, Inc., Berkeley, CA).

### 2.3 fMRI experiment

Participants underwent two separate experimental sessions, 7 days apart (Figure 1). In the first session, participants completed a pre-test (see Section 2.4), the offline behavioral recalibration task (see Section 2.5.1), and reading assessments, lasting approximately 45 min in total. Tasks were performed in a sound-attenuated room, with auditory stimuli delivered through noise-canceling headphones (SONY MDR-7509HD) at a comfortable listening level. The second session involved a mock scanner session to familiarize participants with the scanning environment, complete a second pre-test using MR-compatible headphones (Sensimetrics, model S14, https://www.sens.com) to establish the most ambiguous token for the scanning session, practice reducing head motion while lying in the scanner, and become accustomed to using the MR-compatible button boxes. This second pre-test was conducted without concurrent scanner acquisition noise, using the same MR-compatible headphones later used during scanning, so that the acoustic properties of stimulus presentation were consistent between the pre-test and the scanning session itself. The mock training session took approximately 10 min. Following mock training, participants completed the MRI experiment, during which they performed the same recalibration tasks as in the offline session (i.e., Session 1, outside the scanner), but adapted for the MRI environment. The MRI session lasted 1 h and 15 min, with 45 min dedicated to data acquisition and the remaining time used for positioning participants in the scanner and providing short breaks between tasks.

**Figure 1:**
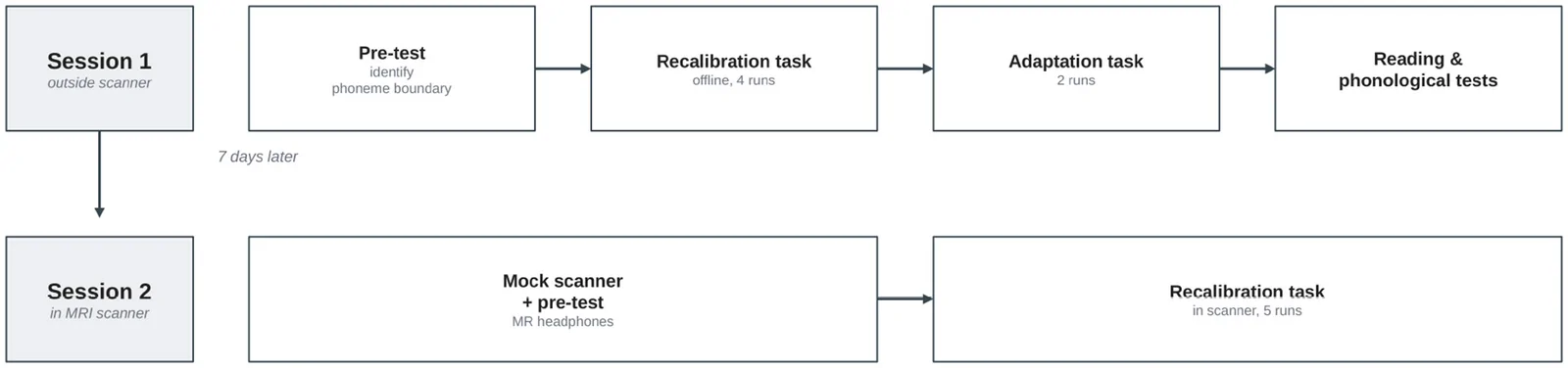
Overview of the experimental design. Session 1 (outside the scanner) comprised the pre-test, the offline recalibration task (4 runs), the adaptation task (2 runs), and the reading and phonological assessments. Session 2 (in the MRI scanner), 7 days later, comprised mock scanner training with a second pre-test, followed by the recalibration task (5 runs). Each recalibration-task run alternated text-based and lipread-based exposure blocks, each followed by auditory post-test trials (see Figure 3 for block/trial structure).

### 2.4 Pre-test

Each participant completed a pre-test twice—once before the offline recalibration task (first session) and once before the scanning recalibration task (second session, during mock training)—to individually establish their most ambiguous stimulus for each session. The most ambiguous sound for each participant was determined separately for each session to account for potential differences between the offline and MRI acoustic environments. The experimental design described below was identical for both pre-test sessions. Participants were presented with nine sound tokens along the /aba/-/ada/ continuum a total of 98 times in a randomized order, with the seven ambiguous tokens presented more frequently than the clear /aba/ and /ada/ sounds to obtain more precise measurements around the individual phoneme boundary where perceptual uncertainty is highest (e.g., Bertelson et al., 2003; Kilian-Hütten et al., 2011b; Vroomen et al., 2004). The frequency of presentation for each stimulus token varied to obtain more precise measurements around each participant's individual phoneme boundary, with the central tokens (3 to 7) presented 14 times, tokens 2 and 8 presented 8 times, and the endpoints (tokens 1 and 9) presented 6 times. Participants were instructed to carefully attend to each sound and respond whether they perceived each sound as /aba/ or /ada/ following a response cue by pressing the left button for /aba/ or the right button for /ada/ (Figure 2). The response cues, displaying “aba” (left) and “ada” (right), were held up by cartoon monsters created with the Monster Workshop content pack of iClone 6 software (https://www.reallusion.com/). During sound presentation, participants viewed a black screen with a white fixation cross, followed by the response cue 1 s later. Each trial ended upon the participant's response, after which the subsequent sound was presented following a 2-s interval. The total duration of the pre-test was approximately 5 min. The most ambiguous speech sound was determined based on the proportion of /aba/ responses to each token along the /aba/-/ada/ continuum and identified as the token with a response proportion closest to 0.5, representing individual participants' perceived phoneme boundary (Romanovska et al., 2019; Vroomen et al., 2004).

**Figure 2:**
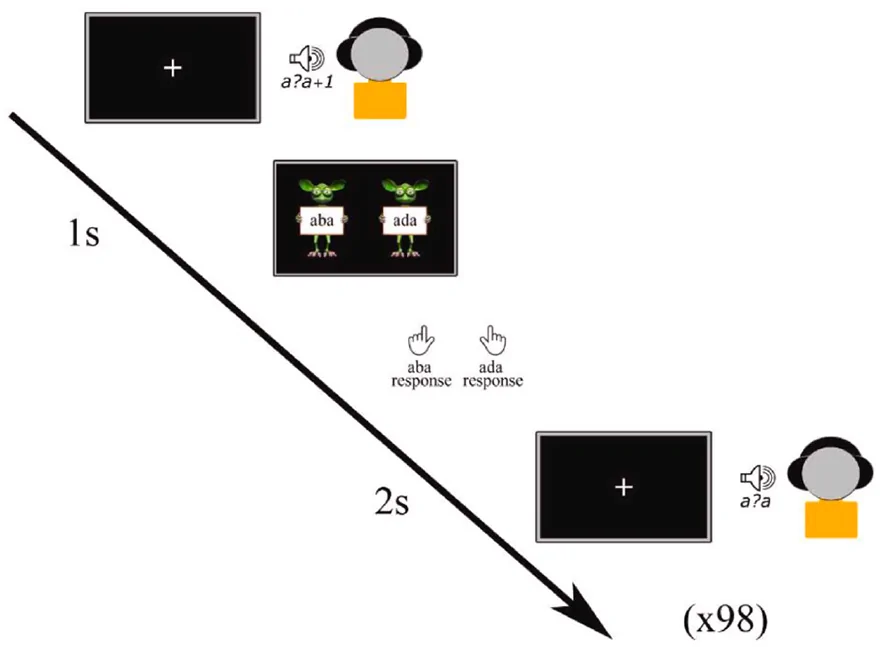
Pre-test. Participants listened to speech sounds along an /aba/-/ada/ continuum while viewing a black screen with a white fixation cross. Following each sound presentation, cartoon monster characters appeared holding response cues displaying “aba” **(left)** and “ada” **(right)**. Participants indicated their perception of each sound by pressing the corresponding left or right button. The individually determined most ambiguous sound (closest to 0.5 response proportion) was used in subsequent recalibration tasks. The cartoon characters were created by the authors using the Monster Workshop content pack of iClone 6 software (Reallusion, https://www.reallusion.com/), used under the software license (adapted).

### 2.5 Offline tasks

#### 2.5.1 Recalibration task

The offline recalibration task was used to obtain behavioral measures of recalibration effects outside the scanning environment. Two types of recalibration paradigms were presented: a text-based and lipread-based recalibration version. Each of them included audio-visual exposure blocks followed by auditory-only post-test trials. Participants completed four 6-min runs, each containing 10 text-based and 10 lipread exposure blocks (with 5 “aba” and 5 “ada” blocks for both text-based and lipread exposure blocks). Following each exposure block, four auditory-only post-test trials were presented, resulting in a total of 40 post-test trials per exposure type for each run. The text and lipread blocks were presented in a pseudo-randomized order, counterbalanced across participants to minimize systematic order effects. In the text exposure blocks, participants viewed eight repetitions of either the text “aba” or “ada” paired with the most ambiguous speech sound /a?a/ (individually determined from the pre-test). During the lipread exposure blocks, the same ambiguous sound was presented together with videos of lip movements articulating “aba” or “ada”. Both the visual and auditory stimuli in the exposure block were presented simultaneously (stimulus onset asynchrony [SOA] = 0 ms), with the sound lasting 650 ms and the text or lip video displayed for 1 s. The 650 ms auditory stimulus was onset-aligned with the lip video; in the lipread condition, the final video frame was held static for the remaining duration to equate visual presentation time (1 s) across the text and lipread conditions. The inter-trial interval (ITI) between subsequent audio-visual exposure trials was 2 s. Participants were instructed to focus on the stimuli without responding. Following each exposure block, four auditory-only post-test trials were presented: the most ambiguous /a?a/ sound was presented twice and each of its flanking sounds (/a?a/+1 and /a?a/-1) were presented once in random order. After each post-test sound, a response cue with “aba” and “ada” texts held by cartoon monsters appeared. Participants were asked to make a forced-choice “aba”/“ada” judgment by pressing the left button for “aba” or the right button for “ada” when the response cue appeared. Responses were self-paced, with no emphasis on speed, and the onset of the response cue was jittered by 1–2 s relative to the post-test sound, with the response cue remaining visible until button press. After the participant's response, there was a 2-second interval before the next post-test trial began.

#### 2.5.2 Adaptation task

After the pre-test and the recalibration task, each participant also completed two runs of an adaptation paradigm. It was identical to the recalibration task except for the audio-visual stimuli, which combined clear /aba/ and /ada/ sounds with corresponding text for congruent stimuli. The adaptation task served to investigate perceptual shifts following exposure to unambiguous speech sounds and to control for potential response biases by comparing performance with clear (adaptation task) vs. ambiguous (recalibration task) auditory stimuli.

All participants completed the recalibration task first, followed by the adaptation task. This fixed order was maintained across all participants to minimize the potential influence of adaptation effects on subsequent recalibration effects (Vroomen and Baart, 2011). More specifically, this order was motivated by the different temporal dynamics of these effects: recalibration effects are typically short-lived and can be disrupted by subsequent auditory exposure, while adaptation effects are more robust and longer-lasting (Vroomen and Baart, 2011; Romanovska et al., 2019).

### 2.6 Recalibration task in the scanner

Participants completed 5 functional runs in total, each containing 4 text-based and 4 lipread exposure blocks (2 “aba” and 2 “ada” blocks for both text-based and lipread exposure blocks). Following each exposure block, four auditory-only post-test trials were presented, resulting in a total of 80 post-test trials per exposure type across all runs. This represents a reduced number of trials relative to the offline version (160 post-test trials per exposure type), consistent with previous fMRI recalibration studies (Bonte et al., 2017; Romanovska et al., 2021) reflecting MRI session time constraints. The recalibration paradigm used in the scanner was similar to the offline version but adapted for the MRI environment (Figure 3). As in the offline version, each exposure block contained eight presentations of either text (“aba” or “ada”) or lipread videos paired with the ambiguous speech sound /a?a/. The text and lipread blocks were pseudo-randomized according to an ABBA and BAAB pattern and counterbalanced across participants and runs to ensure equal presentation frequencies across conditions and to minimize order effects. The scanner implementation differed from the offline version by including a jittered rest period, averaging 10 s (4, 5, or 6 TRs) (compared to no rest period between exposure blocks and post-test trials in the offline version), during which a white fixation cross appeared on a black screen. This jittered rest period served to decorrelate the hemodynamic responses to successive exposure block and post-test trial onsets, improving the GLM's ability to separately estimate the response to each condition. Within the GLM, these implicit fixation rest trials were used to model the baseline. Another difference with the offline paradigm concerned the post-test phase. The response cue onset was jittered between 2.5–3 s after each post-test sound (compared to 1–2 s jittering in the offline version) and lasted for 3 s. The next post-test trial appeared 3–3.5 s after the response cue ended (compared to a fixed 2-s interval in the offline version). This timing maintained a total inter-trial interval of 6 s (3 TR) between consecutive post-test trials (following Romanovska et al., 2021).

**Figure 3:**
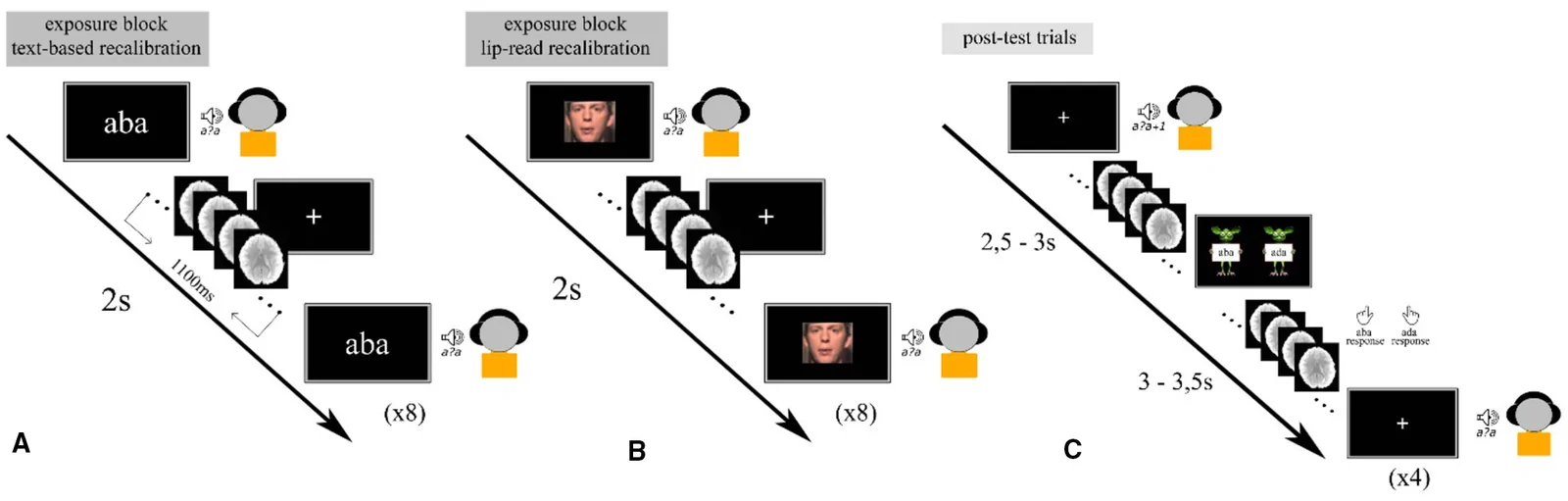
Text-based and lipread recalibration paradigms in the MRI scanner. Exposure blocks of text **(A)** and lipread **(B)** stimuli paired with ambiguous speech sounds (8 stimuli per block), with a 2 s inter-stimulus interval (TR) and a 1,100 ms acquisition period (TA), leaving 900 ms silent gaps for stimulus presentation. In the subsequent post-test trials **(C)** the ambiguous sounds (4 trials per post-test phase), were presented with jittered periods before and after the response cue presentation and the time-window in which the participants provided their response. Also here a TR of 2 s, and a TA of 1,100 ms was used leaving 900 ms silent gaps for post-test sound presentation. The jittered rest period between exposure blocks and post-test trials (averaging 10 s; see Methods) is not separately depicted here. The cartoon characters were created by the authors using the Monster Workshop content pack of iClone 6 software (Reallusion, https://www.reallusion.com/), used under the software license (adapted). The video frames of the speaker are taken from the audiovisual stimulus set of (Bertelson et al. 2003).

### 2.7 MRI measurements

Brain imaging was conducted using a Siemens Prisma 3T MRI scanner (Siemens Medical Systems, Erlangen, Germany) with a 64-channel head-neck coil. Five functional runs, each lasting approximately 5 min, were acquired at a resolution of 2 mm × 2 mm × 2 mm using a multi-band factor of 5 echoplanar imaging (EPI) sequence (repetition time [TR] = 2,000 ms, acquisition time [TA] = 1,100 ms, field of view [FOV] = 210 mm × 210 mm, echo time [TE] = 35.8 ms). Each volume consisted of 60 slices (no gap) covering the whole brain. Speech stimuli were presented binaurally at a comfortable listening level through MR-compatible headphones (Sensimetrics, model S14, https://www.sens.com) during a 900 ms silent gap between consecutive volume acquisitions. Additionally, a high-resolution structural scan (1 mm × 1 mm × 1 mm) was acquired using a T1-weighted three-dimensional MPRAGE sequence ([TR] = 2,300 ms, [TE] = 2.98 ms, 192 sagittal slices).

### 2.8 fMRI pre-processing

Data pre-processing and analysis were conducted using BrainVoyager 23.2 (Brain Innovation, Maastricht, The Netherlands), and custom MATLAB R2022a routines (The MathWorks, Inc., Natick, MA, United States). Functional data underwent 3D motion correction (using trilinear sinc interpolation), slice scan time correction, and high-pass temporal filtering (5 cycles/run). Anatomical data were manually corrected for inhomogeneities to improve white matter-gray matter boundary segmentation and transformed into Talairach space (Talairach and Tournoux, 1988). The functional data were co-registered with the anatomical data, transformed into Talairach space, and spatially smoothed with a 6 mm FWHM Gaussian kernel. Participants with excessive head motion (≥3 mm translation or rotation in any direction) during scanning were excluded from the analysis. For each participant, individual cortical surface representations were automatically created based on the white matter-gray matter boundary and aligned using cortex-based alignment procedure, which iteratively optimizes surface alignment based on cortical curvature patterns to establish anatomical correspondence across participants. This process yielded an anatomically aligned group-average 3D cortical representation (Frost and Goebel, 2012) used for visualization of group-level statistical results. Each participant's functional data were projected onto their individual aligned cortical surface, generating surface-based time courses. All functional data were then analyzed per hemisphere at the surface level. During data acquisition, technical difficulties resulted in 5 participants (2 typical and 3 dyslexic readers) each missing one functional run. These participants were retained in the analysis with their available data.

### 2.9 Statistical analyses of behavioral data

Behavioral performance online (in-scanner) and offline was analyzed separately for text-based and lipread-based recalibration tasks using a 2 x 2 x 3 mixed-design ANOVA with factors Group (2 levels: dyslexic vs. typical readers) × Exposure (2 levels: “aba” vs. “ada”) × Post-test Sounds type (3 levels: /a?a/-1, /a?a/, /a?a/+1). Statistical significance was set at α = 0.05 for all analyses. Mixed-design ANOVAs were conducted on the proportion of /aba/ responses, which was calculated as the number of times participants identified a post-test sound as “/aba/” divided by the total number of responses for that sound condition. Because the /a?a/ sound was presented twice as often as the flanking /a?a/-1 and /a?a/+1 sounds, computing proportions separately within each sound condition accounted for this difference in presentation frequency. The proportion reflects participants' perceptual bias toward one phoneme category, with successful recalibration indicated by higher /aba/ response proportions following “aba” exposure compared to “ada” exposure. Degrees of freedom were adjusted using the Greenhouse-Geisser correction where the sphericity assumption was violated. For significant effects, follow-up pairwise comparisons were conducted to examine specific differences between conditions. *P*-values for multiple comparisons were corrected using the False Discovery Rate (FDR) method to control for Type I errors. For the adaptation task, the same analytical approach was applied, comparing response patterns following exposure to clear /aba/ vs. clear /ada/ sounds paired with corresponding text or lipread information.

### 2.10 fMRI analysis

Cortical activation was assessed using BrainVoyager 23.2's two-level random effects (RFX) general linear model (GLM) analysis. At the first level, GLM analyses were performed separately on each participant's cortical surface, yielding one beta estimate per predictor and voxel. The model included six predictors, related to the “aba” and “ada” text exposure condition, “aba” and “ada” lipread exposure condition, and two predictors for post-test trials following text and lipread exposure blocks. We focused our analysis on the exposure blocks because previous recalibration studies have shown that neural changes during audiovisual learning occur primarily during exposure to paired stimuli, while post-test trials reflect the perceptual outcome rather than the integration process itself (Kilian-Hütten et al., 2011a; Romanovska et al., 2019, 2021).

At the second level, beta estimates from individual participants were entered into random-effects analyses to compute group-level statistical maps for contrasts of interest. We first characterized activation patterns within each condition, followed by targeted between-condition comparisons within predefined regions of interest (ROIs). The contrast of text exposure blocks vs. baseline served to identify brain regions specifically involved in integrating ambiguous speech with orthographic information, isolating neural mechanisms underlying letter-speech sound integration central to reading development. We examined lipread exposure blocks vs. baseline to identify regions involved in integrating ambiguous speech with visual speech information, serving as a control condition to determine whether group differences are specific to orthographic processing or reflect general audiovisual integration difficulties. We also directly compared text vs. lipread exposure blocks to identify regions showing differential activation between the two modalities. For between-group comparisons, we performed whole-brain analyses comparing dyslexic vs. typical readers separately for each exposure condition to identify condition-specific group differences in neural activation patterns. Statistical maps were corrected using a cluster-based threshold determined through 1,000 Monte Carlo simulations to establish the minimum number of contiguous vertices required for a cluster to be considered significant.

In a next step, we functionally defined ROIs by contrasting the beta values (computed at the first stage of the RFX-GLM) of all exposure blocks vs. baseline and cluster-thresholding the resulting statistical map. This contrast provided an approach to ROI selection that captures brain areas generally activated by the task (Frost and Goebel, 2012), without systematic bias of the direction or magnitude of the modality × group interaction subsequently tested within these ROIs. As the ROIs were derived from the present dataset, rather than from an independent localizer task or pre-registered anatomical hypotheses, they are not independent. Consequently, the ROI analysis should be interpreted exploratorily. Importantly, the resulting ROIs did closely converge with those identified in previous fMRI studies employing the same audiovisual recalibration paradigm (Bonte et al., 2017; Kilian-Hütten et al., 2011a, b; Romanovska et al., 2021), including superior temporal, middle temporal, fusiform, and frontal regions consistently implicated in the reading and audiovisual speech network. Once the ROIs were localized, the beta values from each condition were first averaged across each ROI and then used to run a 2 x 2 mixed-design ANOVA with modality (text/lipread) as within-subject factors and group (Typical Readers/Dyslexic Readers) as between-subjects factor performed in SPSS (version 29). Multiple comparisons correction was applied to the ANOVA results using False Discovery Rate (FDR) correction across all ROIs with significance threshold set at *q* = 0.10 (set a priori), using MultipleTesting.com (Menyhart et al., 2021). This significance threshold was chosen to balance sensitivity for detecting subtle audiovisual integration effects while maintaining specificity, consistent with approaches in neuroimaging studies of multisensory processing and whole-brain ROI-based analyses (Ard et al., 2015; Gustison et al., 2024; Schwartzman et al., 2009). The robustness of effects at this threshold was further validated through non-parametric permutation testing (see Supplementary Materials). FDR control was applied across all main effects and interactions from all ROIs. This approach allows for a comprehensive statistical examination of both main effects and interactions between modality and group within functionally defined regions.

To explore potential links between audiovisual integration and behavioral performance, we performed correlation analyses between the extracted beta values within the ROIs during both text and lipread exposure conditions and participants' behavioral measures, including single word reading scores and phonological task performance. These correlations aimed to investigate whether individual differences in neural activation strength during audiovisual integration predict (1) reading abilities and (2) behavioral recalibration magnitude for both text and lipread conditions, calculated from the online (in-scanner) post-test trials.

As sensitivity analysis, non-parametric permutation test was performed to validate the results from the mixed-design ANOVA. Because the design is mixed and includes both a between-subjects factor (Group) and a repeated-measures factor (Modality), permutation testing was constrained to preserve the appropriate statistical unit of exchangeability. For testing the main effect of Group and the Group × Modality interaction, Group labels were permuted across subjects, thereby reassigning subjects to groups while preserving their repeated Modality profiles. This procedure generates datasets consistent with the null hypothesis of “no group effect” and “no interaction effect.” For each permutation, the full ANOVA model was recomputed and the resulting F-values for the main effect of Group and the Group × Modality interaction were stored separately, producing an empirical null distribution against which the observed value was compared. Permutation-based *p*-values were derived from 1,000 resamples. Permutation analyses were conducted in R (version 4.5.2), with FDR correction (*q* = 0.10) applied using built-in R functions. Detailed permutation test results are provided in Supplementary Materials.

## 3 Results

### 3.1 Reading and phonological skills

Descriptive statistics for reading fluency and phonological processing performance are presented in Table 1. For the phonological processing task, accuracy-corrected reaction times that account for both speed and accuracy of performance were compared between the two groups using independent samples *t*-tests. As expected, participants with dyslexia scored significantly lower on the word reading task than typical readers. For the phoneme awareness (PA) task, there was no statistically significant difference between the two groups. However, participants with dyslexia exhibited a trend for a longer accuracy-corrected reaction times than typical readers.

Table 1Descriptive statistics of the sample and group comparisons of dyslexic and typical readers.

**Table:** 

Group	Dyslexic readers	Typical readers	Group difference
Age (SD)	25.17 (6.85)	23.38 (4.91)	
Gender ratio (m/f)	6:12	5:11

**Table:** 

**Measure**	**M**	**SD**	**Range**	**M**	**SD**	**Range**	***t***	***p***
Word reading^1^	78.61	13.06	53–113	97.71	14.71	67–113	−3.89	0.001
Phoneme awareness^2^	96.83	42.70	18.2–212.7	70.00	30.31	37.9–156.9	1.94	0.063

^1^ Number of correct items per 60 s.

^2^ Accuracy-corrected reaction time = total response time/score × 10 (in seconds).

M, mean; SD, standard deviation. *t*-scores assume equal variances for group comparisons. Two typical readers were missing both reading scores, and one was missing phoneme awareness data.

### 3.2 Behavioral recalibration and adaptation results (offline)

Both typical (TpR) and dyslexic (DR) readers showed robust text- and lipread-based recalibration effects outside the MRI scanner, with exposure to ambiguous sounds paired with either “aba” or “ada” text or lip-movement stimuli systematically shifting subsequent perception toward the exposed category (Figure 4). For the text-based condition, the average proportions of /aba/ (vs. /ada/) responses for the most ambiguous sound /a?a/ shifted to 0.67 after exposure to “aba” text and to 0.36 after “ada” text, with proportions in DRs (0.67 vs. 0.37) and TpRs (0.67 vs. 0.35). In the lipread-based condition, the average proportions of /aba/ vs. /ada/ responses after the corresponding videos were 0.80 and 0.18, respectively, again with proportions in DRs (0.82 vs. 0.18) and TpRs (0.78 vs. 0.18).

**Figure 4:**
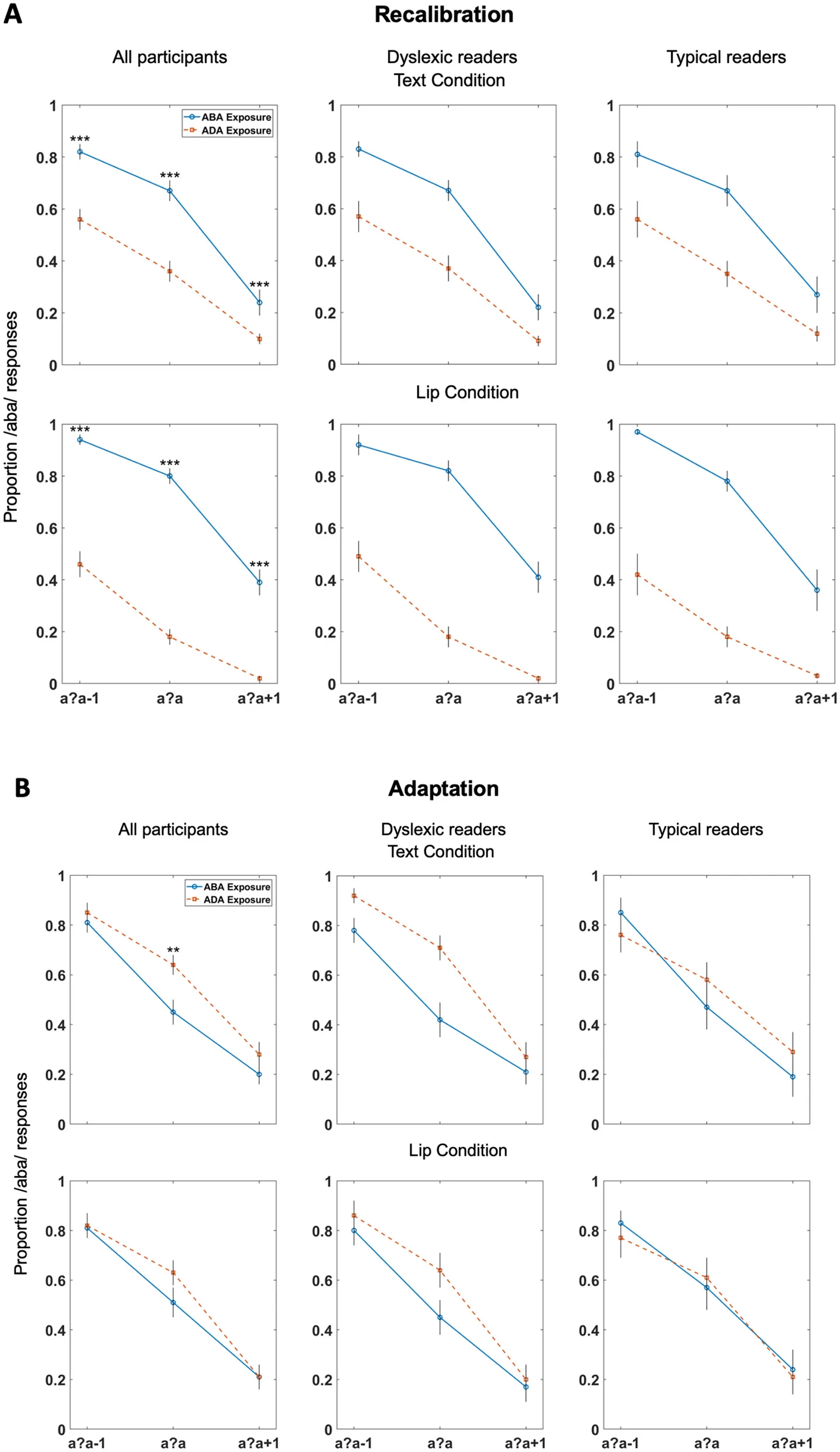
Behavioral recalibration and adaptation results outside the scanner. /aba/ response proportions for post-test sounds /a?a/-1, /a?a/ and /a?a/+1 following “aba” vs. “ada” exposure blocks. On the x-axis, /a?a/-1 is more aba-like and /a?a/+1 is more ada-like. **(A)** Recalibration effects for text-based and lipread-based conditions. **(B)** Adaptation effects for text-based and lipread-based conditions. Statistical significance is indicated only for all participants combined due to the absence of significant Group × Exposure interactions. Vertical bars = standard error; ^**^p ≤ 0.01, ^***^p ≤ 0.001.

Separate 2 (Group: dyslexic vs. typical readers) × 2 (Exposure) × 3 (Post-test Sound) mixed-design ANOVAs on the proportion of /aba/ responses were conducted for the text-based and lipread-based conditions, with Group as a between-subjects factor and Exposure and Post-test Sound as within-subjects factors. No significant group-related effects on recalibration were observed in either condition. Main effects of Exposure (text: *F*(1,32) = 55.36, *p* < 0.001, η^2^*p* = 0.634; lip: *F*(1,32) = 134.05, *p* < 0.001, η^2^*p* = 0.807), indicated that responses differed significantly between “aba” and “ada” exposure conditions. Significant main effects of Post-test Sound (text: *F*(1.69,64) = 97.59, *p* < 0.001, η^2^*p* = 0.753; lip: corrected *F*(1.56,64) = 113.01, *p* < 0.001, η^2^*p* = 0.779), showed that responses varied systematically across the three distinct post-test sounds (/a?a/-1, /a?a/, /a?a/+1) positioned at different ambiguity levels along the /aba/-/ada/ continuum (Figure 4). Most importantly, significant Exposure × Post-test Sound interactions (text: *F*(2,64) = 6.50, *p* = 0.003, η^2^*p* = 0.169; lip: *F*(2,64) = 16.17, *p* < 0.001, η^2^*p* = 0.336), indicated that the magnitude of the recalibration effect varied across the different ambiguity levels of the post-test sounds.

Pairwise comparisons revealed significant recalibration effects across all post-test sounds for both conditions. In the text-based condition, participants gave significantly more /aba/ responses following “aba” exposure than “ada” exposure for: /a?a/-1 (mean difference = 0.26, *SE* = 0.046, *p* < 0.001), /a?a/ (mean difference = 0.31, *SE* = 0.039, *p* < 0.001), and /a?a/+1 (mean difference = 0.15, *SE* = 0.041, *p* = 0.001). For the lipread condition, the corresponding differences were: /a?a/-1 (mean difference = 0.49, *SE* = 0.055, *p* < 0.001), /a?a/ (mean difference = 0.62, *SE* = 0.043, *p* < 0.001), and /a?a/+1 (mean difference = 0.36, *SE* = 0.051, *p* < 0.001). Notably, the recalibration effect was particularly strong for lipread exposure (average effect size = 0.49, calculated as the mean of the three pairwise comparison differences), showing approximately twice the magnitude of perceptual shift compared to text exposure (average effect size = 0.24). This difference is also reflected in the larger partial eta-squared values for the Exposure main effect (lip: η^2^_*p*_ = 0.807 vs. text: η^2^_*p*_ = 0.634), both representing large effects.

Both typical and dyslexic readers showed adaptation effects in the text-based condition, with exposure to clear /aba/ and /ada/ sounds paired with corresponding text shifting subsequent perception of ambiguous sounds away from the clear sound (Figure 4B). For the text-based condition, the average proportions of /aba/ (vs. /ada/) responses to the most ambiguous post-test sound /a?a/ were 0.45 after /aba/ exposure and 0.64 after /ada/ exposure, with patterns in DRs (0.42 vs. 0.71) and TpRs (0.47 vs. 0.58). In the lipread-based condition, the average proportions of /aba/ vs. /ada/ responses were 0.51 and 0.63, respectively, with similar patterns across groups: DRs (0.45 vs. 0.64) and TpRs (0.57 vs. 0.61).

Two separate 2 (Group) × 2 (Exposure) × 3 (Post-test Sounds) mixed-design ANOVAs on the proportion of /aba/ responses confirmed the absence of significant group differences in adaptation for both the text and lip conditions. For the text-based condition, a significant main effect of Exposure (*F*(1,32) = 5.75, *p* = 0.022, η^2^*p*= 0.152) demonstrated a contrastive adaptation effect with participants giving significantly fewer /aba/ responses following “aba” exposure compared to “ada” exposure. A significant main effect of Post-test Sound (*F*(2,64) = 124.03, *p* < 0.001, η^2^*p* = 0.795), showed that response patterns varied systematically across the three distinct post-test sounds (/a?a/-1, /a?a/, /a?a/+1). Additionally, a significant Exposure × Post-test Sound interaction (*F*(2,64) = 3.88, *p* = 0.026, η^2^*p* = 0.108) followed by *post-hoc* analyses showed that the adaptation effect was strongest for the most ambiguous sound /a?a/, where participants gave significantly more /aba/ responses following “ada” exposure compared to “aba” exposure (mean difference = −0.19, *SE* = 0.059, *p* = 0.002, 95% CI [−0.314, −0.074]). No significant differences were observed for the two flanking sounds. In contrast, for the lipread-based condition, no main effect of Exposure was found, suggesting the absence of an adaptation effect. Yet, a main effect of Post-test Sound (*F*(2,64) = 104.25, *p* < 0.001, $\eta_{p}^{2}$ = 0.765), indicated that participants' responses varied systematically, and in the expected way, across the ambiguous sound continuum, with a decrease in the proportion of /aba/ responses from /a?a/-1, to /a?a/, to /a?a/+1 post-test sounds (Figure 4B).

### 3.3 Behavioral results in the scanner

The behavioral results from MRI scanner sessions (*n* = 34) showed a notable reduction in the magnitude of the recalibration effect compared to offline sessions (Figure 5). For the text-based condition, the average proportions of /aba/ responses to the most ambiguous sound /a?a/ shifted to 0.56 after “aba” exposure and to 0.29 after “ada” exposure, with patterns in DRs (0.63 vs. 0.33) and TpRs (0.47 vs. 0.25). In the lipread-based condition, the average proportions of /aba/ responses after the corresponding videos were 0.67 and 0.21, respectively, again with proportions in DRs (0.64 vs. 0.16) and TpRs (0.70 vs. 0.27).

**Figure 5:**
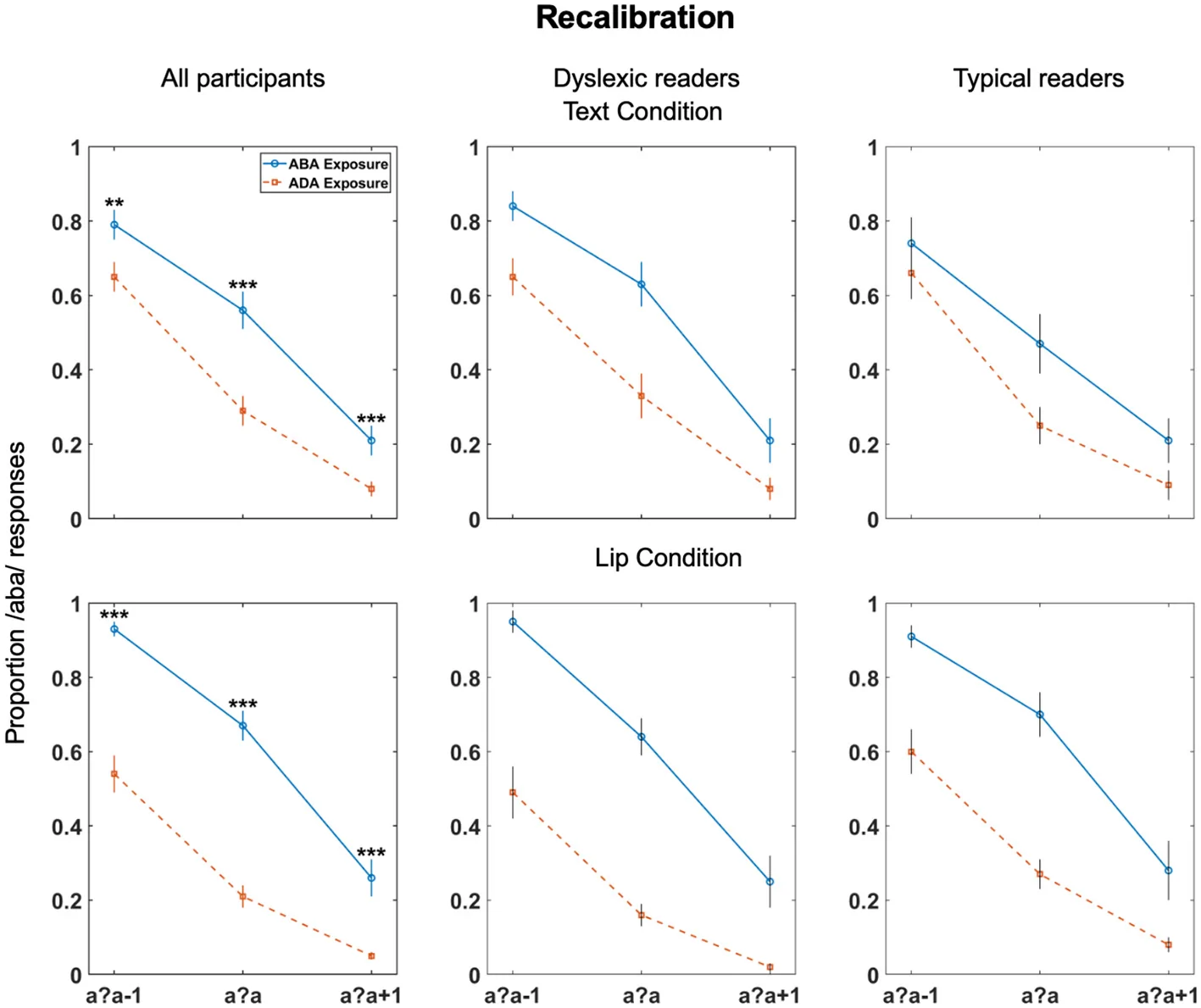
Behavioral text-based and lipread-based recalibration results in the scanner. The graphs show /aba/ response proportions for the /a?a/-1, /a?a/ and /a?a/+1 post-test sounds following an “aba” vs. “ada” exposure block. Statistical significance is indicated only for all participants combined due to the absence of significant Group × Exposure interactions. Vertical bars = standard error; ^**^p ≤ 0.01, ^***^p ≤ 0.001.

Separate 2 (Group) × 2 (Exposure) × 3 (Post-test Sounds) mixed-design ANOVAs on the proportion of /aba/ responses for text-based and lipread-based conditions confirmed the absence of group differences in recalibration for both conditions. Main effects of Exposure (text: *F*(1,32) = 21.12, *p* < 0.001, η^2^*p* = 0.398; lip: *F*(1,32) = 86.88, *p* < 0.001, η^2^*p* = 0.731), indicated that responses differed significantly between “aba” and “ada” exposure conditions. Significant main effects of Post-test Sound (text: *F*(2,64) = 120.98, *p* < 0.001, η^2^*p* = 0.791; lip: corrected *F*(1.55,64) = 172.42, *p* < 0.001, η^2^*p* = 0.843), showed that responses varied systematically across the three distinct post-test sounds (/a?a/-1, /a?a/, /a?a/+1; Figure 5). Significant Exposure × Post-test Sound interactions (text: *F*(2,64) = 6.79, *p* = 0.002, η^2^*p* = 0.175; lip: *F*(2,64) = 14.66, *p* < 0.001, η^2^*p* = 0.314), indicated that the magnitude of the recalibration effect varied across the different ambiguity levels of the post-test sounds.

Pairwise comparisons revealed significant recalibration effects across all post-test sounds for both conditions. In the text-based condition, participants gave significantly more /aba/ responses following “aba” exposure than “ada” exposure for: /a?a/-1 (mean difference = 0.13, *SE* = 0.048, *p* = 0.007), /a?a/ (mean difference = 0.26, *SE* = 0.052, *p* < 0.001), and /a?a/+1 (mean difference = 0.13, *SE* = 0.034, *p* = 0.001). For the lipread-based condition, the corresponding differences were: /a?a/-1 (mean difference = 0.39, *SE* = 0.048, *p* < 0.001), /a?a/ (mean difference = 0.46, *SE* = 0.047, *p* < 0.001), and /a?a/+1 (mean difference = 0.22, *SE* = 0.043, *p* < 0.001).

### 3.4 fMRI activity during audio-visual exposure

During the audio-visual exposure blocks, both text-based and lipread recalibration conditions elicited significant blood-oxygen-level-dependent (BOLD) responses in a broad bilateral network of brain regions commonly associated with reading and audio-visual integration (Bonte et al., 2017; Dehaene et al., 2015; Shaywitz and Shaywitz, 2008; Van Atteveldt et al., 2004). The combined analysis of all exposure blocks vs. baseline was conducted to assess the overall network of brain areas involved and to functionally define regions of interest. This analysis yielded the occipital cortex, left fusiform, bilateral superior temporal gyrus (STG), and frontal and parietal regions (Talairach coordinates listed in Table 2). Given that this combined analysis largely overlapped with the individual condition analyses, we present the condition-specific results below.

**Table 2:** Talairach coordinates of cortical activation clusters during the audio-visual exposure blocks (lipread + text-based recalibration) compared to baseline across groups.

**Area**	Hemisphere	Cluster size (*n* vertices)	Talairach coordinates (center of gravity)		
			x	y	z
Frontal	Left	36	−37	46	8
STG/STS	Left	2,117	−54	−16	7
pMTG	Left	691	−62	−23	8
Lateral	Left	1,181	−58	−9	14
Sensorimotor					
Fusiform	Left	419	−37	−48	−14
Frontal	Right	128	46	31	16
STG/STS	Right	2,379	54	−23	12
pMTG	Right	130	42	−58	11
Parietal	Right	502	31	−44	47
Lateral	Right	885	56	−7	12
Sensorimotor					
Fusiform	Right	253	38	−46	−14

STG, Superior Temporal Gyrus; STS, Superior Temporal Sulcus; pMTG, Posterior Middle Temporal Gyrus.

Separate analyses of text-based exposure blocks vs. baseline (Figure 6) and lipread exposure blocks vs. baseline (Figure 7) revealed largely overlapping activation patterns, with both conditions engaging similar networks involved in audio-visual speech processing. The direct comparison between text-based and lipread exposure conditions revealed minimal differences at the cluster-corrected threshold (FDR < 0.05). Notably, the lipread condition showed stronger activation in the posterior middle temporal gyrus (pMTG) cluster compared to the text-based condition, consistent with previous studies reporting the involvement of middle temporal gyrus regions during audiovisual speech processing (Hall et al., 2005; Kilian-Hütten et al., 2011a,b). The pattern of activated areas during exposure blocks did not show significant group differences between dyslexic and typical readers (Figures 6b,c, and 7b, c). Whole-brain group comparisons did not reveal statistically significant differences between participants with and without dyslexia for the combined all exposure blocks vs. baseline at the FDR < 0.05 level.

**Figure 6:**
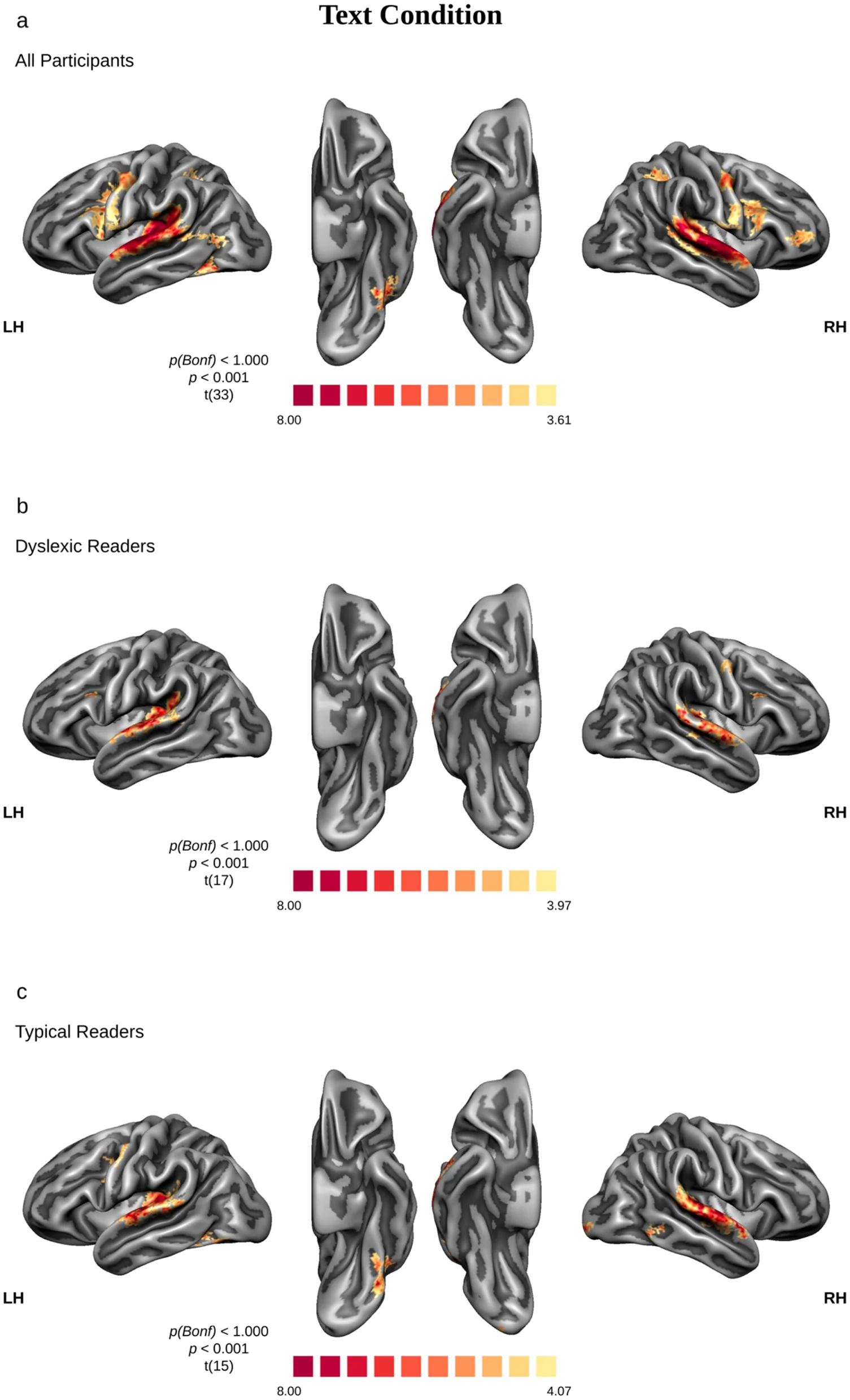
Cortical activation during the exposure blocks of the text-based recalibration condition vs. baseline in **(a)** all participants; **(b)** dyslexic readers; **(c)** typical readers.

**Figure 7:**
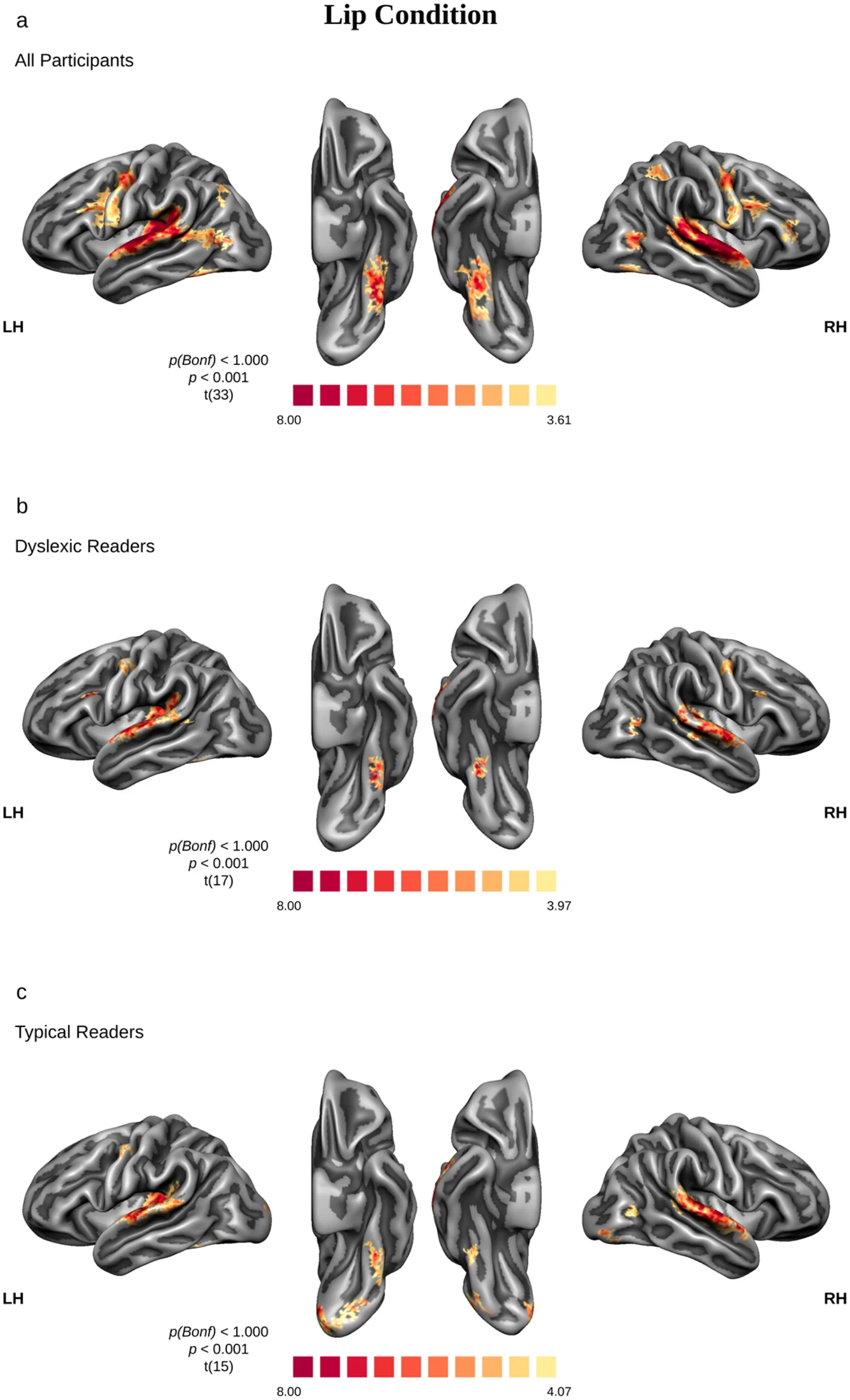
Cortical activation during the exposure blocks of the lipread-based recalibration condition vs. baseline in **(a)** all participants; **(b)** dyslexic readers; **(c)** typical readers.

### 3.5 ROI-based group comparisons and correlations

To investigate group differences in brain regions typically associated with audiovisual processing of text and speech sounds (Blau et al., 2010; Kilian-Hütten et al., 2011a,b; Romanovska et al., 2021), we performed additional ANOVA analyses within functionally defined ROIs. These ROIs were derived from the cluster-thresholded statistical map of all exposure blocks vs. baseline (see Section 2.10 and Table 2) and included: bilateral frontal, bilateral STG/STS, bilateral posterior middle temporal gyrus (pMTG), bilateral lateral sensorimotor, bilateral fusiform, and right parietal regions. For each ROI, we extracted the average beta values across all voxels within that region for each experimental condition (text-based and lipread exposure blocks vs. baseline) and each participant.

Mixed-design ANOVA results revealed varying condition differences across the ROIs during text vs. lipread-based audiovisual exposure blocks. Regions that showed stronger activation during the lipread as compared to text condition included the left posterior MTG (main condition effect *F*(1, 32) = 7.109, *p* = 0.012, η^2^_*p*_ = 0.182; mean difference = 0.038, *p* = 0.012, 95% CI [0.009, 0.067]), the left fusiform region (main condition effect *F*(1, 32) = 6.689, *p* = 0.014, η^2^_*p*_ = 0.173; mean difference = 0.039, *p* = 0.014, 95% CI [0.008, 0.070]), the right posterior MTG (main condition effect *F*(1, 32) = 20.074, *p* < 0.001, η^2^_*p*_ = 0.385; mean difference = 0.09, 95% CI [0.049, 0.130]), and the right fusiform region (main condition effect *F*(1, 32) = 37.452, *p* < 0.001, η^2^_*p*_ = 0.539; mean difference = 0.120, 95% CI [0.080, 0.160]). Both fusiform regions further showed a significant condition × group interaction (left fusiform: *F*(1, 32) = 9.008, *p* = 0.005, η^2^_*p*_ =0.220; right fusiform: *F*(1, 32) = 7.283, *p* = 0.011, η^2^_*p*_ = 0.185). Pairwise comparisons indicated no significant group difference in the text condition, but in the lipread condition, dyslexic readers showed significantly higher activation than typical readers (left fusiform: mean difference = 0.078, *p* = 0.045, 95% CI [0.002, 0.155]; right fusiform mean difference = 0.107, *p* = 0.037, 95% CI [0.007, 0.208]).

Significant condition × group interactions were also observed in the right frontal area (*F*(1, 32) = 7.641, *p* = 0.009, η^2^_*p*_ = 0.193). In the right frontal area, TpRs showed a higher fMRI response during text than the lipread condition (mean difference = 0.031, *p* = 0.033, 95% CI [0.003, 0.060]), while no significant difference was observed for DRs. Permutation testing confirmed the robustness of these ROI-based statistical results (detailed results provided in Supplementary Materials).

Correlation analysis showed a moderately positive correlation between phonological test performance (accuracy-corrected reaction times (RTs) in the Spoonerism task) and activation in the lipread condition within the left fusiform ROI (*r*(29) = 0.484, *p* = 0.006) (Figure 8A), indicating that higher activation was associated with poorer phonological skills (longer RTs). When examined by group, this correlation was significant specifically within the dyslexic readers (*r*(16) = 0.540, *p* = 0.021), while no significant correlation was observed in typical readers (*r*(11) = 0.042, *p* = 0.893). Moreover, activation in the text condition within the right posterior MTG positively correlated with Spoonerism RT scores (*r*(29) = 0.390, *p* = 0.030) (Figure 8B). When examined by group, neither dyslexic readers nor typical readers (*ps* > 0.05) showed significant correlations individually. No significant correlations were found between single word reading scores and activation strength in any of the ROIs for either exposure condition.

**Figure 8:**
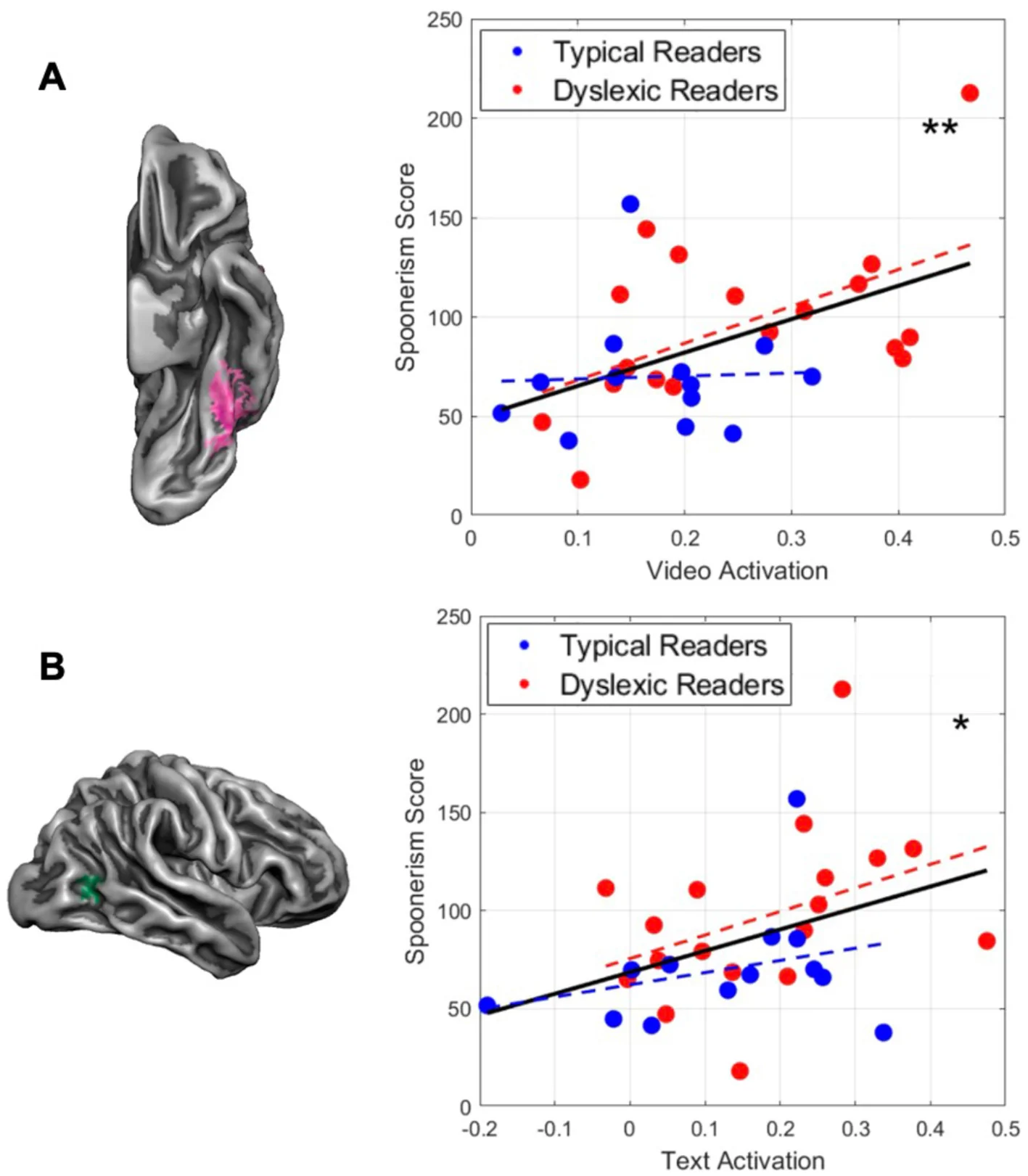
Results of the correlation analyses between cortical activation during the audio-visual exposure blocks within the ROIs and phonological test scores: **(A)** left fusiform region, **(B)** right posterior middle temporal gyrus. X-axis: BOLD signal change (% signal change relative to baseline); Y-axis: Spoonerism task accuracy-corrected RT (in seconds).

## 4 Discussion

The present fMRI study examined text-based and lipread-based recalibration in adults with and without developmental dyslexia. Our findings show no significant group differences in behavioral recalibration effects and broadly similar patterns of neural activation during audiovisual exposure blocks in both groups. Both groups showed greater MTG and fusiform activation during lipread compared to text conditions, reflecting the enhanced visual speech processing demands of lipreading. Subtle but significant group differences included increased bilateral fusiform activation during lipread exposure in Dyslexic readers. Additionally, left fusiform activation during lipread exposure correlated with individual phonological processing abilities as measured with Spoonerisms task performance.

### 4.1 Behavioral findings

Our behavioral results demonstrated robust recalibration effects in both typical and dyslexic readers, with no significant group differences in either text-based or lipread-based recalibration. This aligns with findings by Romanovska et al. (2019, 2021) in children with dyslexia, who showed comparable text-based recalibration to typical readers. However, it contrasts with work by Keetels et al. (2018), who reported a selective reduction for text-based but not lipread-based recalibration in adults with dyslexia. Several methodological differences may contribute to this discrepancy. First, our paradigm employed separate blocks for text-based and lipread-based recalibration, whereas Keetels et al. (2018) intermixed these conditions within blocks. When mixing the conditions, attention to the two modalities could have been differentially influenced, potentially masking text-based recalibration effects; we note this as a possible methodological explanation rather than a conclusion drawn from our data. Indeed, our fMRI results show stronger engagement of bilateral fusiform regions during lipread exposure blocks in dyslexic readers compared to typical readers. Second, our participant groups were more heterogeneous in terms of age and educational background compared to the more homogeneous samples in Keetels et al. (2018), which may have influenced the detection of overall group differences in audiovisual integration abilities.

Consistent with previous literature (Keetels et al., 2016, 2018; Bonte et al., 2017; Romanovska et al., 2019, 2021), our results showed that lipread-based recalibration had larger effect sizes than text-based recalibration. This is likely due to the more natural and direct correspondence between visually perceived articulatory movements and speech sounds compared to the more arbitrary mapping between letters and phonemes (Van Atteveldt et al., 2004; Blomert, 2011). This may partly reflect the strong biological grounding of lip-speech associations, which infants can already exploit shortly after birth without explicit training (Kuhl and Meltzoff, 1982), in contrast to letter-speech sound mappings that are only acquired later through explicit literacy instruction. Furthermore, the reduced magnitude of recalibration effects observed in the MRI environment compared to offline session likely reflects the impact of scanner noise, posture constraints, and attentional factors on audiovisual integration processes. Romanovska et al. (2021) reported even stronger reductions in recalibration effects within the scanner environment for children (i.e., no significant behavioral text-based recalibration effects at the group level in the scanner), suggesting that this environmental sensitivity spans developmental stages and may be particularly challenging for paradigms requiring subtle perceptual judgments.

To examine potential unisensory auditory adaptation effects and response biases (Samuel, 1986; Samuel and Kraljic, 2009; Vroomen and Baart, 2011), in our offline behavioral experiment, we included non-ambiguous exposure blocks where participants were exposed to clear /aba/ and /ada/ tokens paired with congruent text or lipread information. While text-based adaptation showed the expected significant contrastive effects, lipread-based adaptation did not show significant adaptation effect. We expected significant adaptation effects for both conditions given that selective adaptation found to represent a unisensory auditory phenomenon (Eimas and Corbit, 1973; Vroomen et al., 2004, 2007), occurring following exposure to clear speech tokens regardless of accompanying visual information (Vroomen et al., 2007) including text and lipread videos (Keetels et al., 2016, 2018). Importantly, the adaptation blocks in the lipread condition also showed no significant effect in the opposite direction, confirming that the recalibration effect we observed was not purely due to response bias (e.g., responding /aba/ just because of having seen ‘aba' before), which strengthens the validity of our findings as representing genuine perceptual shifts rather than strategic response patterns (van Linden and Vroomen, 2008; Romanovska et al., 2019).

### 4.2 Neural activation during exposure blocks

Our fMRI analyses focused on neural activity during the audiovisual exposure blocks, which reflects the audiovisual integration process itself, rather than on the post-test trials, which instead reflect its perceptual (recalibration) outcome (Kilian-Hütten et al., 2011a; Romanovska et al., 2019, 2021).

Based on our initial hypotheses, we predicted that dyslexic adults would show reduced text-based recalibration accompanied by reduced left fusiform gyrus activation and increased left STG activation during text-based exposure blocks (Keetels et al., 2018; Romanovska et al., 2021). Lipread-based recalibration was not expected to differ significantly between groups, with additional MTG recruitment in both groups as compared to text-based recalibration. Our findings partially align with these predictions but also reveal unexpected results.

Overall, dyslexic and typical readers activated a similar network of cortical areas during audiovisual exposure blocks across both the text-based and lipread-based conditions. This network included the bilateral superior temporal gyri (STG), ventral occipito-temporal cortex (vOTC), and frontal regions, aligning with previous studies on letter-speech sound integration and audiovisual speech processing (Blau et al., 2010; Bonte et al., 2017; Van Atteveldt et al., 2004; Romanovska et al., 2021; Ye et al., 2017). The activated brain regions during our audiovisual exposure blocks largely overlap with those reported in previous fMRI studies of text-based (Bonte et al., 2017) and lipread-based recalibration (Kilian-Hütten et al., 2011a,b) in typically reading adults, suggesting that the core audiovisual integration network is consistently engaged across different visual modalities and populations. Specifically, lipread compared to text conditions showed the expected increased activation in the posterior middle temporal gyrus/cortex (pMTG) (Bonte et al., 2017; Kilian-Hütten et al., 2011a,b). The pMTG is known to play a critical role in biological motion processing and is specifically engaged during the perception of facial movements during speech, serving as a key region for integrating visual speech information with corresponding auditory signals (Calvert et al., 2000; Campbell, 2008).

The absence of significant group differences in the whole-brain analysis suggests that adults with dyslexia engage largely similar neural circuitry during the processing of ambiguous speech sounds combined with text or videos with lipread speech information. Our exploratory ROI analyses did reveal more subtle differences between groups. Contrary to our prediction of reduced left fusiform activation during text-based processing in dyslexic readers, we found no group differences in the text condition. Instead, dyslexic readers showed significantly higher bilateral fusiform activation specifically during the lipread condition. This region is consistently implicated in both face perception (Kanwisher et al., 1997; Grill-Spector et al., 2004) and orthographic processing, containing the so-called visual word form area (VWFA) (Dehaene et al., 2015). In particular, previous studies have suggested that the fusiform gyrus contains specialized yet partially overlapping neural populations for processing faces and written words, with both types of visual stimuli requiring high-resolution foveal processing and expertise (Dehaene and Cohen, 2011). Our observed group effect may thus specifically relate to neural differences in audiovisual speech processing during the extraction of lipread information in the exposure blocks. Alternatively, the increased fusiform activation might relate to more general group differences in face or biological-motion processing. Although the latter cannot be excluded, our finding of a positive correlation between left fusiform activation during the lipread condition and Spoonerisms test performance (longer RTs) indicates that higher activation was associated with poorer phonological processing skills, would be more consistent with a language/speech related interpretation. For example, individuals with weaker phonological processing abilities may allocate greater attention to visual speech cues (lipreading) to disambiguate ambiguous auditory speech information (Pekkola et al., 2006; MacSweeney et al., 2009). This interpretation aligns with previous findings showing that individuals with dyslexia may rely more heavily on visual speech information, particularly under challenging listening conditions such as speech in noise (Baart et al., 2012; De Gelder and Vroomen, 1998). Keetels et al. (2018) demonstrated that while adults with dyslexia showed reduced text-based recalibration, their lipread-based recalibration remained intact, suggesting preserved or even enhanced lipread speech processing. This pattern differs from findings in children by Romanovska et al. (2021), who found positive correlations between left fusiform activation in the text-based condition and better reading fluency and phoneme deletion skills, suggesting that the relationship between fusiform activation and phonological abilities may be both modality-specific and developmentally dynamic.

While we predicted increased left STG activation during text-based processing in dyslexic readers, our results showed a condition × group interaction in the right STG/STS region that was significant at the uncorrected level but did not survive our FDR (*q* = 0.1) correction across ROIs, where dyslexic readers showed higher activation during lipread compared to text conditions, and typical readers showed no difference between conditions. Although this finding should be interpreted with caution, an increased activation in the right STG/STS region among dyslexic readers during lipread compared to text conditions would be consistent with the general pattern reported by Romanovska et al. (2021), who found that increased STG activation during audiovisual processing was associated with less fluent letter-speech sound identification in children. This increased right STG/STS activation may reflect the application of the principle of inverse effectiveness, where multisensory integration is enhanced when individual sensory inputs are relatively weak (Stevenson and James, 2009). While the visual stimuli in our paradigm are clear, the auditory stimuli are intentionally ambiguous, which could be considered “weaker” in terms of sensory input. The visual information (lipread speech) might therefore be utilized to facilitate auditory stimulus processing, increasing neural interaction and improving stimulus identity prediction (Van Atteveldt et al., 2014). In dyslexic readers, this facilitation might require additional neural resources due to less automatized audiovisual integration processes, resulting in the observed increase in right-lateralized auditory cortical activation. While the laterality and modalities differ, both studies (current data, Romanovska et al., 2021) suggest altered processing patterns in superior temporal regions during audiovisual integration in individuals with dyslexia.

Dyslexic and typical readers further engaged the right frontal areas somewhat differently depending on the type of visual input (text vs. lipread information). Typical readers showed higher activation in right frontal areas during the text-based audiovisual exposure blocks. While there is no clearcut explanation for this finding, the functional significance of this difference could be further elucidated through connectivity analyses or examination of event-related response patterns, which represent interesting avenues for future analysis of the current dataset (Van Atteveldt et al., 2014). Given that behavioral performance did not differ significantly between groups, these activation differences likely reflect alternative processing strategies rather than processing deficiencies.

### 4.3 Limitations and future directions

Several limitations should be considered when interpreting our findings. First, our sample size of 18 DRs and 16 TpRs, while comparable to previous fMRI studies on phonetic recalibration (Bonte et al., 2017; Kilian-Hütten et al., 2011a,b; Romanovska et al., 2021), may limit the statistical power to detect subtle group differences. Accordingly, in our data analysis we selected an FDR correction of *q* = 0.1 a priori to detect subtle but consistent effects across both parametric ANOVA and non-parametric permutation testing. Notably, the significant condition × group interactions we did detect were associated with medium-to-large effect sizes (left fusiform: η^2^*p* = 0.220; right fusiform: η^2^*p* = 0.185), suggesting adequate sensitivity to detect effects of this magnitude despite the modest sample size. Future studies with larger samples could provide more definitive evidence regarding the neural correlates of audiovisual integration in dyslexia. Second, the cross-sectional nature of our study prevents us from drawing conclusions about the developmental trajectory of audiovisual integration in dyslexia. Longitudinal studies tracking the development of these neural systems from childhood to adulthood would provide valuable insights into how compensatory mechanisms emerge and evolve over time. Third, the reduced magnitude of recalibration effects in the MRI environment compared to offline behavioral sessions highlights the challenge of studying subtle perceptual phenomena in the scanner. Future studies might benefit from more extensive familiarization with the scanning environment or alternative experimental designs that are less susceptible to scanner-related interference. Fourth, our study focused primarily on cortical activation patterns during audiovisual exposure blocks. Future research could extend these findings by examining functional connectivity between regions involved in letter-sound integration, which may reveal more nuanced differences in how information flows through these neural networks in dyslexic vs. typical readers. Additionally, investigating how the magnitude of behavioral recalibration effects scales with fMRI activity during exposure blocks would provide important insights into the relationship between neural activation strength and perceptual plasticity, potentially revealing whether stronger neural responses predict greater behavioral recalibration (and adaptation) in audiovisual speech processing. Fifth, the absence of a significant group difference in phoneme awareness may partly reflect a ceiling effect given the limited item pool of the Spoonerisms task (20 items), combined with our high-functioning, university-recruited dyslexic sample; the trend toward longer accuracy-corrected reaction times in the dyslexic group nonetheless points in the expected direction. Future studies incorporating a dynamic, motion-matched non-speech control condition (e.g., mouth movements not corresponding to recognizable phonemes, or temporally scrambled/reversed lip videos) would help dissociate face- and motion-related contributions from speech-specific contributions to the fusiform effect reported above. Our behavioral analysis also aggregated post-test responses across all blocks of a given exposure type rather than modeling block-level carryover effects (e.g., transition type or trial position). Because block order was fully counterbalanced across participants, such carryover effects are unlikely to have systematically biased our fMRI or behavioral results, though explicitly modeling them could further reduce noise in future analyses. Finally, an exploratory analysis correlating fusiform activation during the lipread condition with individual differences in behavioral recalibration magnitude assessed in the scanner did not reveal significant associations (left fusiform: *r* = 0.13, *p* = 0.45; right fusiform: *r* = 0.03, *p* = 0.88), suggesting that a direct brain-behavior link, if present, may require a larger sample to detect.

### 4.4 Conclusion

Our study provides novel insights into the neural mechanisms underlying audiovisual processing of letter-sound and lipread-sound associations in adults with and without developmental dyslexia. While both groups showed no significant differences in behavioral recalibration effects, subtle differences in neural activation, particularly in the left fusiform area during lipread-based audiovisual processing and its correlation with phonological skills, indicate possible group differences in neural activation during lipread-based audiovisual processing, persisting into adulthood. These findings suggest group differences in neural engagement specifically when integrating ambiguous speech sounds with visual lipreading information, rather than with text-based visual input. Future research should continue to explore how these neural systems develop and adapt over time, potentially informing more targeted interventions for individuals with reading difficulties across the lifespan.

## Data Availability

The raw data supporting the conclusions of this article will be made available by the authors upon reasonable request.
