# Supplementary material for: Cortical responses to lipread and text-based audiovisual stimuli in adults with and without dyslexia
Source: Front Hum Neurosci. 2026 Oct 23;20:1806964. doi: 10.3389/fnhum.2026.1806964 (PMC13739575; doi:10.3389/fnhum.2026.1806964)
Supplement: Supplementary file 1 [file Data_Sheet_1.pdf]

Supplementary Materials

1. Non-Parametric Permutation Testing Analysis

Permutation testing largely replicated the parametric ANOVA results (Table S1). After FDR correction, significant main effects of condition were observed in four regions (left posterior MTG, bilateral fusiform, and right posterior MTG), consistent with differential cortical responses to text versus lip-read phonetic recalibration. Significant group × condition interactions emerged in three regions (left fusiform, right frontal area, and right fusiform), suggesting group-dependent modality effects. No main effects of group survived FDR correction.

Table S1. Significant Effects from Non-Parametric Permutation Testing

Brain Region	Effect	*F*-statistic	*p*-value (FDR-corrected)
Left posterior MTG	Condition	8.22	0.033
Left fusiform	Condition	7.66	0.052
Left fusiform	Group × Condition	9.01	0.033
Right frontal area	Group × Condition	7.64	0.046
Right posterior MTG	Condition	20.51	< 0.001
Right fusiform	Condition	39.56	< 0.001
Right fusiform	Group × Condition	7.28	0.052
